# Ethnic inequalities in acute myocardial infarction and stroke rates in Norway 1994–2009: a nationwide cohort study (CVDNOR)

**DOI:** 10.1186/s12889-015-2412-z

**Published:** 2015-10-20

**Authors:** Kjersti S. Rabanal, Randi M. Selmer, Jannicke Igland, Grethe S. Tell, Haakon E. Meyer

**Affiliations:** Division of Epidemiology, Norwegian Institute of Public Health, P.O. Box 4404, Nydalen, 0403 Oslo Norway; Department of Global Public Health and Primary Care, University of Bergen, P.O. Box 7804, N-5018 Bergen, Norway; Department of Health Registries, Norwegian Institute of Public Health, Kalfarveien 31, 5018 Bergen, Norway; Department of Community Medicine, Institute of Health and Society, University of Oslo, P.O. Box 1130, Blindern, 0318 Oslo Norway

**Keywords:** Acute myocardial infarction, Cardiovascular disease, CVDNOR, Immigrants, Ethnicity, Stroke

## Abstract

**Background:**

Immigrants to Norway from South Asia and Former Yugoslavia have high levels of cardiovascular disease (CVD) risk factors. Yet, the incidence of CVD among immigrants in Norway has never been studied. Our aim was to study the burden of acute myocardial infarction (AMI) and stroke among ethnic groups in Norway.

**Methods:**

We studied the whole Norwegian population (*n* = 2 637 057) aged 35–64 years during 1994–2009. The Cardiovascular Disease in Norway (CVDNOR) project provided information about all AMI and stroke hospital stays for this period, as well as deaths outside hospital through linkage to the Cause of Death Registry. The direct standardization method was used to estimate age standardized AMI and stroke event rates for immigrants and ethnic Norwegians. Rate ratios (RR) with ethnic Norwegians as reference were calculated using Poisson regression.

**Results:**

The highest risk of AMI was seen in South Asians (men RR = 2.27; 95 % CI 2.08–2.49; women RR = 2.10; 95 % CI 1.76–2.51) while the lowest was seen in East Asians (RR = 0.38 in both men (95 % CI 0.25–0.58) and women (95 % CI 0.18–0.79)). Immigrants from Former Yugoslavia and Central Asia also had increased risk of AMI compared to ethnic Norwegians. South Asians had increased risk of stroke (men RR = 1.26; 95 % CI 1.10–1.44; women RR = 1.58; 95 % CI 1.32–1.90), as did men from Former Yugoslavia, Sub-Saharan Africa and women from Southeast Asia.

**Conclusions:**

Preventive measures should be aimed at reducing the excess numbers of CVD among immigrants from South Asia and Former Yugoslavia.

**Electronic supplementary material:**

The online version of this article (doi:10.1186/s12889-015-2412-z) contains supplementary material, which is available to authorized users.

## Background

Europe has become a multi-ethnic continent with increasing migration across borders. Ethnic minority and migrant populations consequently make up substantial proportions of European populations [1]. The immigrants in Europe are heterogeneous in relation to age, sex, country of birth, socioeconomic status, type of migration, and they also vary in risk of cardiovascular diseases (CVD) [2]. In Norway overall, approximately 13 % of the population are immigrants compared to 32 % in the capital Oslo [3]. A large proportion of these immigrants comes from developing countries where the rates of CVD are rapidly increasing [3, 4]. Immigrants from South Asia (countries such as Pakistan, Sri Lanka, India and Bangladesh) have a higher risk of coronary heart disease (CHD) as compared to local populations and other immigrant groups in the United Kingdom (UK), Denmark and Sweden [5–8]. Increased risk of CHD in South Asians in other parts of the world has also been reported [9, 10], suggesting a possible underlying susceptibility for CHD in this group. South Asian immigrants are prone to diabetes and metabolic disturbances such as abdominal adiposity, dyslipidaemia and hyperglycaemia [11], this has also been documented among South Asians in Norway [12, 13]. Still, the burden of CVD among this immigrant group is currently unknown.

Few studies have assessed the risk of CVD among immigrants from Former Yugoslavia (including countries such as Croatia, Slovenia, Bosnia-Hercegovina, Macedonia, Serbia, Montenegro and Kosovo) settled in Western European countries. Previous studies from Denmark and Sweden report no marked differences in incidence of CVD between Former Yugoslavian immigrants and the native populations [6, 8]. A more recent Swedish study, however, found higher incidence of first time acute myocardial infarction (AMI) in male immigrants from Former Yugoslavia compared to native Swedes [14]. A recent Danish study also found higher risk of CHD among immigrants from Former Yugoslavia compared to native Danes [15]. According to a Framingham risk calculator, immigrants from Former Yugoslavia in Norway have been found to have increased predicted 10-year risk of CVD compared to other ethnic groups [16]. Whether the predicted risk reflects actual risk of disease in this immigrant group is currently unknown.

The incidence of CVD among immigrants in Norway has never been reported. This nationwide study aimed to describe the burden of acute myocardial infarction and stroke among immigrants in Norway, compared to ethnic Norwegians.

## Methods

### Cardiovascular disease in Norway: the CVDNOR project

The CVDNOR project contains CVD hospitalization data for the whole Norwegian population for the period 1994–2009. Hospital stays with ICD9 codes 390–459 or ICD10 codes I00-I99 were extracted from the Patient Administrative Systems in all Norwegian somatic hospitals from 1994 to 2009 (www.cvdnor.no). The database includes information on age, sex, dates of hospitalization and discharge, main and secondary diagnoses, procedures, departments, wards, time of transfers between departments/wards and type of hospitalization. It has been linked to The Cause of Death Registry, and The Population Registry containing demographic and socioeconomic data for all subjects. Further details on this database are given elsewhere [17, 18].

Due to the young age distribution among immigrants in Norway, we included individuals aged 35–64 years (*N* = 2 652 123) at risk of having an AMI or stroke during 1994–2009. Country of birth was used to identify immigrants (born abroad with at least one parent born abroad). We therefore excluded persons with missing information on country of birth (*n* = 1 310), and individuals with a foreign country of birth whose parents were both born in Norway (*n* = 13 746). Some small countries were also excluded (St. Helena (*n* = 5), the British Indian Ocean Territory (*n* = 1), the Maldives (*n* = 2) and the Falkland Islands (*n* = 2)), leaving a total sample of 2 637 057 individuals for analyses. The population at risk was updated January 1^st^ each year during 1994–2009. We grouped the immigrants into 14 larger regions (see Additional file 1: Table A1). Countries of birth with sufficient numbers were also analyzed individually in addition to the regions.

We identified hospitalizations with AMI (ICD9: 410; ICD10: I21, I22) or stroke (ICD9: 430, 431, 434, 436; ICD10: I60, I61, I63, I64) as main or secondary diagnosis and deaths outside hospital with AMI or stroke as underlying cause of death. For each individual, we included up to 3 events. However, a few individuals contributed with more than 3 events (maximum 6 events) if they had at least 7 event-free years between their third and fourth event. Most of the individuals experienced only one event (88 % of the individuals with AMI and 80 % of the individuals with stroke) during the study period, and 99.9 % experienced ≤ 3 events (both endpoints separately). Additional events were excluded to reduce the possibility of counting events more than once. For the same reason, we only included events with stroke as secondary diagnosis when the main diagnosis was other than rehabilitation. Hospitalizations or deaths occurring ≤ 28 days after a previous hospitalization were considered part of the previous event.

### Statistical analyses

AMI and stroke event rates were calculated using the number of events (numerator) divided by the number of person-years from the population at risk during 1994–2009 (denominator). Persons aged 35–64 contributed with one person-year to the denominator every year they were registered (on January the 1^st^) as Norwegian residents. Age-standardized AMI and stroke event rates with 95 % confidence intervals (CIs) were computed using the direct standardization method, [19] stratified by ethnic group and expressed per 100 000 person-years. The Norwegian population of year 2001 was used as standard population and 5-year age strata were used for the standardization.

Poisson or negative binomial regression analyses (when goodness of fit test for the Poisson model was significant) were used to compute rate ratios (RRs) enabling us to control for calendar year to account for time trends in AMI and stroke. Ethnic Norwegians was the reference group and we adjusted for age in 5-year age groups and for calendar year as indicator variable. All analyses were performed in Stata 13.

#### Sensitivity analyses

We repeated the Poisson regression analyses including only 1 event during the whole period to see whether it influenced the estimates.

In addition to the main analyses, we have also calculated AMI and stroke event rates for a wider age group; 35–89 (see Additional file 1: Tables A2 and A3).

#### Attributable fractions

We calculated the attributable fractions (AF) for groups with increased risk of AMI and stroke (immigrants from South Asia and Former Yugoslavia) using the following formula: AF = (RR-1)/RR [20]. The AFs indicate how much the event rates would have been reduced if the immigrant group had the same risk as ethnic Norwegians. RRs from the Poisson regression model were used in the calculation.

### Ethical considerations

The project was approved by the Regional Committee for Medical Research Ethics, Health Region West.

## Results

During 1994–2009, 1 348 744 women and 1 288 313 men aged 35–64 resided in Norway. Immigrants from 14 different regions totalled 282 485 subjects (45 % women), which constituted approximately 11 % of the study sample.

During the study period, we observed 67 683 AMI events in 59 314 individuals (20 % women) of whom 3 726 were immigrants. Correspondingly for stroke, we observed 43 252 events in 34 392 individuals (37 % women) whereof 2 078 were immigrants.

### Acute myocardial infarction

In Table 1, we show age-standardized AMI event rates for regions and countries of birth. The overall crude AMI rates were 389 per 100 000 person-years in men and 101 per 100 000 person-years in women. Men from all regions had higher standardized rates than their female counterparts, and for most regions this gender difference was 3-fold. For most of the ethnic groups this gender difference was statistically significant (the CIs did not overlap), whereas for three small groups (China, Central America and Oceania/Pacific) the confidence intervals were wide and overlapping.

**Table 1:** Age standardized AMI event rates per 100 000 person-years, subjects aged 35–64 years, CVDNOR 1994–2009

	Men, *n* = 1 288 313			Women, *n* = 1 348 744		
Country or region of birth	N	AMIs	SER (95 % CI)	N	AMIs	SER (95 % CI)
Norway	1 194 414	50469	385 (382–388)	1 160 158	12891	98 (96–100)
Western Europe	56 603	1361	339 (321–357)	45 521	262	67 (59–75)
Denmark	10 581	314	352 (313–391)	9 474	54	56 (41–71)
Finland	3 248	99	434 (348–519)	3 889	20	63 (35–90)
Sweden	12 417	303	348 (308–387)	11 285	74	79 (61–97)
The Netherlands	2 938	46	259 (183–334)	2 221	9	55 (19–91)
Great Britain	9 955	226	300 (261–339)	5 511	49	93 (67–119)
Germany	8 339	178	357 (305–409)	6 552	29	53 (34–73)
Eastern Europe	23 031	220	376 (324–427)	14 550	42	67 (46–88)
Poland	15 698	105	357 (275–440)	5 599	21	67 (37–98)
Russia	1 429	13	349 (145–553)	4 188	3	29 (0–62)
Hungary	832	48	399 (276–521)	571	9	139 (46–231)
Former Yugoslavia	9 805	374	549 (491–606)	8 763	94	176 (140–213)
Bosnia-Hercegovina	4 437	196	537 (461–614)	4 470	57	176 (130–222)
Kosovo	2 790	82	869 (646–1092)	2 130	18	255 (112–398)
Middle East	15 710	402	513 (456–571)	9 445	48	123 (86–160)
Turkey	3 651	114	510 (409–610)	2377	22	166 (93–239)
Iraq	5 323	110	581 (454–709)	2759	9	94 (25–164)
Iran	4 882	127	438 (345–530)	3 245	11	80 (31–130)
North Africa	4 078	55	233 (167–299)	1 803	5	47 (0–95)
Morocco	2 260	32	210 (133–287)	1 198	3	50 (00–113)
Sub-Saharan Africa	10 497	126	259 (206–312)	7 052	17	98 (49–147)
Somalia	3 583	50	405 (265–545)	2 490	6	142 (28–255)
South Asia	13 063	771	812 (752–871)	10 238	121	216 (176–257)
Sri Lanka	3 623	120	707 (550–863)	2 834	6	46 (2–90)
India	2 447	99	514 (411–616)	1 911	17	163 (84–243)
Pakistan	6 115	538	978 (894–1061)	4 967	95	283 (224–342)
Southeast Asia	6 280	102	253 (202–305)	14 304	31	49 (30–69)
Philippines	1 227	30	344 (219–469)	4 642	10	53 (15–92)
Vietnam	4 303	62	223 (164–283)	4 161	8	32 (9–54)
East Asia	2 775	22	165 (94–235)	3 460	7	43 (11–75)
China	1 763	13	137 (62–213)	1 987	5	64 (8–120)
Central Asia	1 347	34	733 (461–1005)	1 195	8	218 (65–371)
North America	5 812	72	226 (173–279)	5 867	18	50 (27–73)
USA	5 025	64	228 (171–284)	5 012	10	31 (12–51)
Central America	710	11	267 (111–424)	1 032	6	140 (21–259)
South America	3 870	84	302 (233–371)	4 342	22	86 (48–125)
Chile	2 472	67	328 (242–413)	1 999	10	63 (22–103)
Oceania/Pacific	749	6	255 (49–462)	583	2	50 (0–120)

*AMI* acute myocardial infarction, *SER* standardized event rate; *CI* confidence interval

**Fig. 1:**
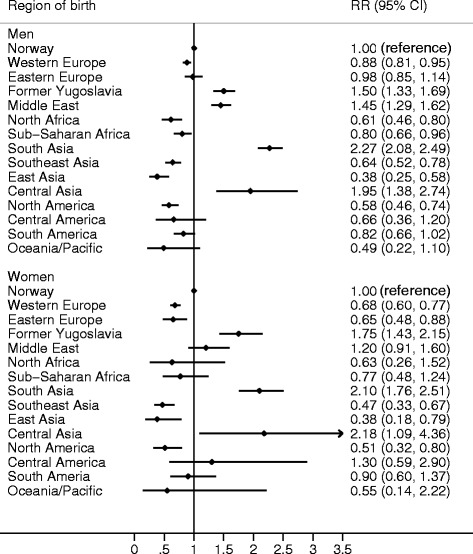
Forest plot showing incidence rate ratios for AMI events in subjects aged 35–64 years. The rate ratios are adjusted for age and calendar year

RRs for AMI, adjusted for age and calendar year, are shown in Fig. 1. Compared to ethnic Norwegians, immigrants from South Asia had the highest risk of AMI which was more than 2-fold in both men and women.

Immigrants from Central Asia had comparable AMI risk as the South Asians, but the CIs for the estimates were wide demonstrating uncertainty.

Immigrant men from Former Yugoslavia and the Middle East had around 50 % increased risk compared to Norwegian men, and immigrant women from Former Yugoslavia had a 75 % increased risk compared to ethnic Norwegian women.

Among countries of birth within South Asia (Sri Lanka, India and Pakistan), immigrants from Pakistan had the highest event rates of AMI. Men from Sri Lanka and India also had high rates compared to ethnic Norwegians (Table 1).

East Asian immigrants had the lowest risk of AMI with a RR of 0.38 for both men and women (Fig. 1). Immigrants from North America, Western Europe, and Southeast Asia, and immigrant women from Eastern Europe also had lower risk of AMI compared to the local population in Norway. Immigrants from North Africa and Sub-Saharan Africa had reduced risk of AMI, although not statistically significant in women.

### Stroke

In Table 2, we show age-standardized rates of stroke for regions and countries of birth. The overall crude stroke rates were 193 per 100 000 person-years in men and 116 per 100 000 person-years in women.

**Table 2:** Age standardized stroke event rates per 100 000 person-years, subjects aged 35–64 years, CVDNOR 1994–2009

	Men, *n* = 1 288 313			Women, *n* = 1 348 744		
Country or region of birth	N	Strokes	SER (95 % CI)	N	Strokes	SER (95 % CI)
Norway	1 194 414	25528	194 (191–196)	1 160 158	15112	116 (115–118)
Western Europe	56 603	715	180 (166–193)	45 521	394	102 (92–112)
Denmark	10 581	186	206 (176–236)	9 474	103	109 (87–130)
Finland	3 248	74	340 (262–418)	3 889	49	155 (112–199)
Sweden	12 417	169	199 (169–229)	11 285	93	100 (80–120)
The Netherlands	2 938	27	159 (98–219)	2 221	12	72 (31–113)
Great Britain	9 955	107	145 (117–172)	5 511	48	95 (68–122)
Germany	8 339	81	161 (126–196)	6 552	45	88 (62–114)
Eastern Europe	23 031	86	157 (123–192)	14 550	76	110 (84–136)
Poland	15 698	36	145 (90–200)	5 599	43	148 (101–196)
Russia	1 429	6	177 (22–331)	4 188	15	77 (33–120)
Hungary	832	30	215 (131–299)	571	5	87 (9–165)
Former Yugoslavia	9 805	158	270 (227–313)	8 763	71	127 (96–157)
Bosnia-Hercegovina	4 437	78	231 (179–283)	4 470	52	151 (109–192)
Kosovo	2 790	24	275 (153–398)	2 130	11	188 (59–316)
Middle East	15 710	133	192 (154–230)	9 445	51	127 (88–165)
Turkey	3 651	42	211 (143–279)	2 377	13	76 (29–123)
Iraq	5 323	49	248 (163–332)	2 759	20	273 (141–404)
Iran	4 882	32	148 (87–209)	3 245	14	103 (47–159)
North Africa	4 078	26	125 (74–176)	1 803	4	32 (0–66)
Morocco	2 260	10	76 (26–126)	1 198	3	33 (0–74)
Sub-Saharan Africa	10 497	111	251 (197–304)	7 052	23	83 (44–122)
Somalia	3 583	49	464 (306–622)	2 490	7	84 (9–159)
South Asia	13 063	214	242 (208–276)	10 238	117	199 (161–238)
Sri Lanka	3 623	33	208 (116–299)	2 834	14	114 (45–182)
India	2 447	39	201 (137–265)	1 911	13	120 (52–188)
Pakistan	6 115	135	264 (219–309)	4 967	86	250 (194–306)
Southeast Asia	6 280	74	176 (134–218)	14 304	124	179 (144–214)
Philippines	1 227	16	180 (90–269)	4 642	43	171 (111–230)
Vietnam	4 303	48	167 (117–218)	4 161	46	183 (128–237)
East Asia	2 775	31	250 (162–339)	3 460	16	76 (38–115)
China	1 763	21	227 (130–324)	1 987	6	55 (9–100)
Central Asia	1 347	6	125 (18–232)	1 195	8	259 (75–442)
North America	5 812	39	122 (83–161)	5 867	40	110 (75–144)
USA	5 025	36	128 (85–170)	5 012	37	119 (80–158)
Central America	710	6	139 (27–250)	1 032	9	182 (53–310)
South America	3 870	42	182 (124–239)	4 342	34	115 (74–157)
Chile	2 472	34	206 (132–280)	1 999	15	104 (50–159)
Oceania/Pacific	749	2	60 (0–144)	583	2	55 (0–132)

*SER* standardized event rate, *CI* confidence interval

As for AMI, men had generally higher rates of stroke compared to women, although this was not true for immigrants from Southeast Asia, Central Asia and Central America, where women had similar rates as their male counterparts.

**Fig. 2:**
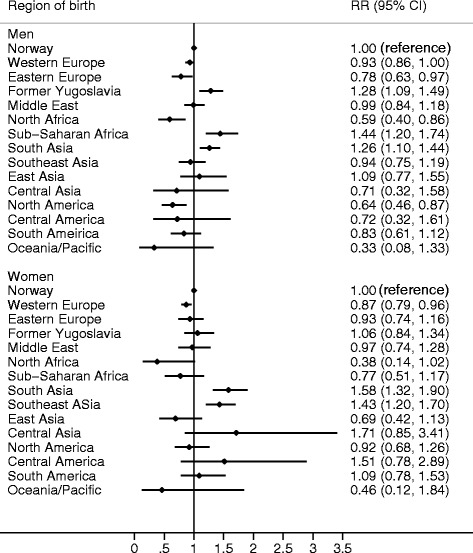
Forest plot showing incidence rate ratios for stroke events in subjects aged 35–64 years. The rate ratios are adjusted for age and calendar year

RRs for stroke, adjusted for age and calendar year, are shown in Fig. 2. In general, the ethnic differences in stroke risk were less consistent across genders compared to the differences in risk of AMI. For example, men from Former Yugoslavia and men from Sub-Saharan Africa had significantly higher risk of stroke compared to ethnic Norwegians (RRs of 1.28; 95 % CI 1.09–1.49 and 1.44; 95 % CI 1.20–1.74 respectively) but women from these regions did not have higher risk.

Immigrants from South Asia formed the only group with increased risk of stroke in both genders.

Reduced risk of stroke was seen in immigrant men from North Africa and North America. Slightly reduced risk was also observed in Eastern European men and Western European women.

### Attributable fractions

If South Asians had the same risk as ethnic Norwegians, their risk would have been 52.4 % and 55.9 % lower than their observed risk, corresponding to a reduction of 63 out of 121 and 431 out of 771 cases of AMI (in women and men respectively) during the 16-year study period. In immigrants from Former Yugoslavia, the corresponding fractions were 42.9 % (representing 40 out of 94 AMI cases) in women and 33.3 % (representing 125 out of 374 AMI cases) in men. The AFs for stroke were 36.7 % in South Asian women and 20.6 % in South Asian men. For Former Yugoslavian men, the AF for stroke was 21.9 %. We did not calculate the AF for stroke in women from Former Yugoslavia since we did not find increased risk of stroke in this group.

### Sensitivity analyses

The sensitivity analyses including only 1 AMI or stroke event per person had little influence on the estimates.

We found similar risk patterns for AMI in the wider age group, 35–89, as we did in our main analyses (see Additional file 1: Table A2). For stroke, the risk pattern was somewhat different when including the wider age group. Among men, immigrants from Eastern Europe constituted the only group with significantly increased risk of stroke (according to the 95 % confidence intervals) compared to ethnic Norwegians (see Additional file 1: Table A3). Among women, immigrants from Former Yugoslavia had significantly increased risk of stroke compared to ethnic Norwegians, and immigrants from South Asia had an excess risk that was borderline significant according to the confidence intervals.

## Discussion

This is the first study to describe the burden of CVD among immigrants in Norway. Our study showed that ethnic groups vary in risk of AMI and stroke, and identified differences in absolute and relative risk. Particularly immigrants from South Asia and Former Yugoslavia were found to have increased risk of AMI compared to other ethnic groups. Despite the relatively young population, we found high numbers of attributable cases in these two immigrant groups. The high numbers illustrate potential benefits from prevention in these high-risk groups. When compared to ethnic Norwegians, immigrants from Western Europe, North America, East Asia and Southeast Asia had reduced risk of AMI, both men and women. Only immigrants from South Asia had increased risk of stroke in both men and women.

Immigrants from South Asia had the highest risk of AMI, more than two-fold compared to ethnic Norwegians. They also had increased risk of stroke. This corresponds well with previous Norwegian studies reporting high levels of cardiovascular risk factors among South Asian immigrants [13, 16, 21, 22]. It was also concordant with the UK literature reporting a particularly high risk of CHD and a higher risk of stroke in immigrants from South Asia compared to the general UK population [5, 7, 23]. While elevated risk of CHD in South Asian populations has been documented in several countries around the world [24], the risk of stroke in this immigrant group has received less focus, especially outside the UK. Within the UK, however, immigrants from South Asia have been found to have increased risk of stroke compared to the native European population in England and Wales, but not in Scotland [25, 26]. The latter possibly due to high stroke rates in the white Scottish comparison population. South Asians come from a region with a high prevalence of stroke, especially in the urban areas. It has been stated that South Asia probably contributes to more than 40 % of the worlds’ stroke related deaths [27]. This fraction is, however, somewhat uncertain, since there is a general lack of population-based studies on the occurrence of stroke in this region [27]. Moreover, most of the available studies are conducted in India and might not be generalizable for the whole region.

The increased risk of CVD in South Asians is not fully understood, but differences in metabolic risk factors have been found to account for some of their excess risk [7, 10]. A recent prospective study from the UK found that waist-to-hip ratio was the individual risk factor that best attenuated the increased risk of CHD in South Asians compared to Europeans, although the risk remained significantly elevated also after adjustment (SHR 1.45, 95 % CI: 1.28–1.64) [7]. With regard to stroke, the same study found that diabetes was associated with a 2.5-fold age-adjusted incidence of stroke in South Asian immigrants.

Former Yugoslavia and Eastern Europe are two geographically close regions. Yet we found that immigrants from these two regions had very different risk of CVD. While immigrants from Former Yugoslavia had elevated risk of both AMI and stroke (the latter in men only) compared to ethnic Norwegians, the immigrants from Eastern Europe had similar or even reduced risk of both cardiovascular endpoints. This difference in risk might be related to differences in selection through migration. Concerning immigrants from Former Yugoslavia, increased risk of CVD could be related to traumatic war experiences prior to migration, since a great proportion of Former Yugoslavian immigrants came as refugees from the Balkan wars in the 1990’s [28]. Posttraumatic stress disorder is associated with increased risk of CVD [29], and psychosocial factors constitute an important risk factor for myocardial infarction and stroke [30, 31]. Immigrants from Eastern European countries are, to a greater extent, labor migrants and may therefore be a healthier group compared to the general population in their home countries. This would be in accordance with the “healthy immigrant effect” hypothesis [32]. Studies addressing the healthy immigrant phenomenon in Europe have, however, found mixed results [32, 33]. One of these studies grouped all immigrants into one group and compared them with the native populations of their host countries [33]. This has its limitations since different immigrant groups often vary in health, as demonstrated in the present study. Also, the healthy immigrant effect might not apply equally to all immigrant groups. The healthy immigrant effect is, for example, not evident in refugees [32]. In our study, lower risk was observed in immigrants from North America and Western Europe. This reduced risk could potentially, to some extent, be explained by the healthy immigrant effect since the reasons for migration for these groups are often related to work, family or education [34].

Another explanation for the healthy immigrant effect is the phenomenon of unhealthy remigration, also known as the “salmon bias” [35]. The salmon bias refers to a compulsion to die in ones birthplace, and is expected to be more pronounced among older immigrants, since they often experience more health problems than the young. Although originally proposed for mortality data, the salmon effect is also relevant for morbidity data. Since we cannot rule out the possibility that immigrants in our study have experienced AMIs or strokes when visiting their home countries, the salmon effect could potentially contribute to an underestimation of AMI and stroke rates. The investigation of the salmon bias has, however, so far been scarce and the documentation of an existing effect is ambiguous [35–37]. A recent European study examining emigration from Denmark found, in fact, *lower* probability of emigration for immigrants with severe diseases [36].

The high risk of CVD found in immigrants from Former Yugoslavia is in accordance with high levels of cardiovascular risk factors previously reported in a Norwegian study for this group [16]. Studies from Sweden and Switzerland have also reported high levels of cardiovascular risk factors in Former Yugoslavian immigrants compared to the native populations, especially concerning overweight and obesity [38–40]. Available information on CVD mortality and morbidity in Former Yugoslavian countries also indicate high rates compared to Western European countries [41, 42]. Only a few studies have reported the incidence of AMI among immigrants from Former Yugoslavia settled in Western European countries, and the findings are somewhat inconclusive [6, 8, 43]. A case–control study from Austria reported increased risk of AMI in young (≤40 years) immigrants from Former Yugoslavia compared to native Austrians [43]. Meanwhile, a register-based study in Denmark did not find increased risk of CVD in this immigrant group compared to native Danes. The women from Former Yugoslavia did, however, have increased risk in some adjusted models [6]. All estimates in the Danish study were adjusted for marital status. In the present study, we have only adjusted for age and calendar year. Thus, a lack of social support indicated by marital status could possibly explain some of the discordance between the two studies. A more likely explanation, however, relates to the fact that the Danish study did not include war refugees. Consequently, the Former Yugoslavian group in the Danish study differed from our Former Yugoslavian group in a way that could have influence their risk of CVD.

As discussed, we found the highest risk of AMI in South Asians, and interestingly, the lowest risk was also observed in immigrants from Asia. Immigrants from East Asia had the lowest risk of AMI and Southeast Asians the second lowest risk. This concur with the literature reporting lower burdens of CHD in East Asian compared to Western populations, but not a lower burden of stroke [44]. The latter also confirmed in our study.

African Caribbean immigrants in the UK have reduced risk of CHD and increased risk of stroke compared to the European UK population [7]. We found decreased risk of AMI and increased risk of stroke in immigrant men from Sub-Sahara African countries concordant with UK findings.

In this study, we focused on a relatively young population regarding CVD risk. Consequently, our results concern the risk of getting CVD in an early age. In agreement with our findings, studies have found that South Asians acquire AMI in earlier ages than other ethnicities [30, 45]. Also, the previously mentioned study from Austria reporting increased risk of AMI in young immigrants from Former Yugoslavia [43] corresponds with this.

The mechanisms underlying ethnic differences in CVD are complex, and to explain the causes of the observed differences in CVD rates is beyond the scope of this paper. Numerous studies have tried to find explanations for the increased risk of CVD in South Asian populations, but so far, it is still not clear how much can be attributed to genetic and/or environmental factors [46]. Referring to the different stages of the epidemiologic transition, we know that CVD rates are dynamic and can be influenced by societal, demographic and environmental changes [47].

### Strengths and limitations

This study has several strengths. First, the large sample size and national coverage make the findings relevant for the whole population in Norway in this age range. Also, the large sample size made it possible to analyse some countries of birth individually. This is a strength because of the heterogeneity in aggregated ethnic groups [48].

By using register data we minimize possible selection bias, although selection bias related to different use of health care services in immigrant groups [49] could possibly be present. We expect this to be limited, however, since we have focused on serious conditions and also included CVD deaths outside hospital. By updating the population at risk every year, we took possible emigration into account. Only immigrants with a valid personal ID were included in this study, thereby excluding individuals currently seeking asylum, tourists and some guest workers [50].

The AMI diagnosis in hospital discharge data in Norway have not been validated, but studies from Denmark and the Netherlands indicate a positive predictive value of about 90 % when AMI is coded as the main diagnosis [51–53]. Incident stroke discharge diagnosis was validated for a region in central Norway for the period 1994–1996 using a population-based stroke register as “gold-standard” [54]. The discharge data were found to overestimate the incidence of stroke, but the validity improved when restricting to acute stroke diagnoses. In the present study we have only used acute diagnoses for both endpoints and have also made other restrictions to reduce possible overestimation such as using the 28-day rule when defining events (see the methods section) and restricting the number of events per person. Also, since overdiagnosis and wrong coding of incident strokes happen more often when stroke is the secondary diagnosis [55], we excluded strokes coded as secondary diagnosis when the main diagnosis was rehabilitation. In a Danish study, AMI coded as secondary diagnosis had only slightly poorer validity, and the combination of National Hospital Registry data and National Death Registry data were found to be valid for monitoring CVD in the Danish population [53]. The validity of both the AMI and stroke diagnoses is unlikely to differ across the ethnic groups, and thus, it is unlikely that the validity of endpoints may have had any influence on the observed ethnic differences in CVD.

## Conclusions

This study identified ethnic differences in risk of AMI and stroke in the Norwegian population aged 35–64 years. In particular, immigrants from South Asia and Former Yugoslavia had increased risk of AMI and stroke compared to ethnic Norwegians. Immigrants from North Africa, Western Europe, Eastern Europe and North America had similar or reduced risk compared to ethnic Norwegians.

This study has identified ethnic groups that should be targeted in future prevention efforts in order to reduce social health inequalities in Norway.

## Additional file

Additional file 1: Table A1. Regions and countries of birth. Norwegian residents aged 35–64, 1994–2009. **Table A2.** Age standardized AMI event rates per 100 000 person-years, subjects aged 35–89 years, CVDNOR 1994–2009. **Table A3.** Age standardized stroke event rates per 100 000 person-years, subjects aged 35–89 years, CVDNOR 1994–2009. The additional tables provide supplementary information to the article. Table A1 lists all the countries within each region. Table A2 and A3 respectively show AMI and stroke event rates for a wider age group than the one we focused on in the article. (PDF 1020 kb)

## Abbreviations

AF: Attributable fraction
AMI: Acute myocardial infarction
CHD: Coronary heart disease
CI: Confidence interval
CVD: Cardiovascular disease
CVDNOR: The cardiovascular disease in Norway project
ICD: International classification of diseases
RR: Rate ratio
UK: United Kingdom
