# Supplementary material for: Ethnic inequalities in acute myocardial infarction and stroke rates in Norway 1994–2009: a nationwide cohort study (CVDNOR)
Source: BMC Public Health. 2015 Oct 20;15:1073. doi: 10.1186/s12889-015-2412-z (PMC4612407; doi:10.1186/s12889-015-2412-z)
Supplement: Additional file 1: Table A1. — Regions and countries of birth. Norwegian residents aged 35–64, 1994–2009. Table A2. Age standardized AMI event rates per 100 000 person-years, subjects aged 35–89 years, CVDNOR 1994–2009. Table A3. Age standardized stroke event rates per 100 000 person-years, subjects aged 35–89 years, CVDNOR 1994–2009. The additional tables provide supplementary information to the article. Table A1 lists all the countries within each region. Table A2 and A3 respectively show AMI and stroke event rates for a wider age group than the one we focused on in the article. (PDF 1020 kb) [file 12889_2015_2412_MOESM1_ESM.pdf]

Table A1 Regions and countries of birth. Norwegian residents aged 35-64, 1994-2009

Regions	Countries
**Norway** (n=2 354 572)	Norway
**Western Europe** (n=102 124)	Denmark (n=20 055), Greenland (n=168), Finland (n=7 137), Fareo islands (n=795), Sweden (n=23 702), Belgium (n=757), Andorra (n=4), France (n=2 972), Gibraltar (n=7), Greece (n=686), Ireland (n=569), Italy (n=1 640), Malta (n=48), Netherlands (n=5 159), Liechtenstein (n=12), Luxembourg (n=29), Monaco (n=4), Portugal (n=631), San Marino (n=4), Spain (n=1 607), Great Briatin (n=15 466), Switzerland (n=1 031), Germany (n=14 891), Austria (n=936), Israel (n=451), Cyprus (n=106), Iceland (n=3 257)
**Eastern Europe** (n=37 581)	Estonia (n=636), Bulgaria (n=1 066), Belarus (n=267), Latvia (n=711), Poland (n=21 297), Romania (n=1 403), Lithuania (n=2 456), Moldavia (n=110), Russia (n=5 617), Ukraine (n=844), Hungary (n=1 403), Slovakia (n=853), Georgia (n=105), Czech Republic (n=813)
**Former Yugoslavia** (n=18 568)	Albania (n=142), Croatia (n=1 693), Slovenia (n=111), Bosnia-Hercegovina (n=8 907), Macedonia (n=1 296), Serbia (n=1 325), Montenegro (n=174), Kosovo (n=4 920)
**Middle East** (n=25 155)	Turkey (n=6 028), Armenia (n=99), Aserbadsjan (n=142), Bahrain (n=8), The United Arab Emirates (n=11), Iraq (n=8 082), Iran (n=8 127), Jordan (n=113), Kuwait (n=102), Lebanon (n=1 075), Palestine (n=639), Qatar (n=3), Saudi Arabia (n=35), Syria (n=636), Yemen (n=52), Oman (n=3)
**North Africa** (n=5 881)	Tunisia (n=604), Algeria (n=823), Egypt (n=475), Libya (n=91), Morocco (n=3 458), Sudan (n=362), Southern Sudan(n=68)
**Sub-Saharan Africa** (n=17 549)	Angola (n=149), Botswana (n=37), Equatorial Guinea (n=3), Ivory Coast (n=116), Eritrea (n=1 644), Ethiopia (n=1 733), Djibouti (n=8), Gambia (n=767), Ghana (n=1 072), Guinea (n=39), Guinea-Bissau (n=10), Cameroon (n=165), Cape Verde (n=350), Congo (n=520), Liberia (n=267), Madagascar (n=168), Mauritania (n=11) Mauritius (n=154), Namibia (n=50) Nigeria (n=518), Mozambique (n=83), Zimbabwe (n=132), Rwanda (n=182), São Tomé and Príncipe (n=1), Senegal (n=73), Central African Republic (n=2), Sierra Leone (n=208), Somalia (n=6 073), South Africa (n=649), Burundi (n=221), Comoros (n=4), Benin (n=8), Gabon (n=7), Congo-Brazzaville (n=46), Kenya (n=586), Lesotho (n=4), Malawi (n=59), Mali (n=23), West-Sahara (n=4), Niger (n=12), Réunion (n=6), Seychelles (n=11), Swaziland (n=6), Chad (n=15), Togo (n=55), Tanzania (n=611), Uganda (n=496), Zambia (n=171), Burkina Faso (n=20)
**South Asia** (n=23 301)	Bangladesh (n=395), Bhutan (n=13), Myanmar (n=740), Sri Lanka (n=6 457), India (n=4 358), Nepal (n=256), Pakistan (n=11 082)
**Southeast Asia** (n=20 584)	Brunei (n=4), Phillipines (n=5 869), Indonesia (n=477), Cambodia (n=165), Laos (n=46) Malaysia (n=326), East-Timor (n=5), Singapore (n=275), Thailand (n=4 953), Vietnam (n=8 464)
**East Asia** (n=6 235)	Taiwan (n=137), Hongkong (n=665), Japan (n=879), China (n=3 750), North-Korea (n=18), South-Korea (n=698), Mongolia (n=24), Macao (n=64)
**Central Asia** (n=2 542)	Afghanistan (n=1980), Kasakhstan (n=351), Tadsjikistan (n=29), Turkmenistan(n=25), Kirgisistan(n=45), Uzbekistan (n=112)
**North America** (n=11 679)	Canada (n=1 642), USA (n=10 037)
**Central America** (n=1 742)	Cayman Islands (n=2), Costa Rica (n=62), Cuba (n=335), Dominica (n=9), the Dominican Republic (n=190), Grenada (n=14), Guadeloupe (n=4), Haiti (n=13), Honduras (n=54), Jamaica (n=91), Martinique (n=9) Mexico (n=397), Aruba (n=19), Curacao (n=42), Nicaragua (n=64), Panama (n=27), El Salvador (n=75), Saint Lucia (n=3), Saint Vincent and Grenadine (n=2), Trinidad and Tobago (n=272), American Virgin Islands (n=2), British Virgin Islands (n=1), Barbados (n=17), Antigua and Barbuda (n=2), Belize (n=8), Bahamas (n=7), Bermuda (n=5), Puerto Rico (n=16)
**South America** (n=8 212)	Guatemala (n=89), Argentina (n=483), Bolivia (n=132), Brazil (n=1 138), Guyana (n=74), Chile (n=4 471), Columbia (n=619), Ecuador (n=166), French Guyana (n=1), Paraguay (n=45), Peru (n=519), Surinam (n=23), Uruguay (n=160), Venezuela (n=292)
**Oceania/Pacific** (n=1 332)	American Samoa (n=1), Australia (n=931), Salomon Islands (n=2), Cook islands (n=4), Fiji (n=24), French Polynesia (n=3), Tonga (n=5) Tuvalu (n=1), New Zealand (n=346), Federated states of Micronesia (n=1), Samoa (n=2), New Caledonia (n=6), Papua New Guinea(n=5), Palau (n=1)

Table A2 Age standardized AMI event rates per 100 000 person-years for subjects aged 35-89 years, CVDNOR 1994-2009

	Men 35-89 years, n=1 634 520			Women 35-89 years, n=1 682 539		
Country or region of birth	N	AMIs	SER (95 % CI)	N	AMIs	SER (95 % CI)
Norway	1 472 970	156129	992 (987-997)	1 542 459	95142	441 (438-444)
Western Europe	***59 931***	***2768***	***1009 (965-1054)***	***51 919***	***1486***	***367 (348-385)***
Denmark	11 830	986	1156 (1073-1239)	11 004	403	363 (326-400)
Finland	3 384	174	1397 (1125-1669)	4 148	70	337 (252-422)
Sweden	13 191	526	937 (843-1031)	13 139	384	380 (342-419)
The Netherlands	3 062	104	1052 (785-1320)	2 416	37	283 (187-379)
Great Britain	10 268	340	824 (700-948)	6 591	245	395 (345-444)
Germany	8 664	300	908 (781-1035)	7 390	198	370 (318-423)
Eastern Europe	***23 801***	***545***	***1216 (1102-1329)***	***15 324***	***154***	***371 (309-433)***
Poland	16 083	252	1243 (1067-1418)	5 804	51	316 (217-415)
Russia	1 499	36	1181 (783-1579)	4 396	23	374 (217-530)
Hungary	938	115	1079 (831-1326)	675	39	541 (360-722)
Former Yugoslavia	***10 370***	***556***	***1167 (1019-1316)***	***9 582***	***269***	***702 (603-801)***
Bosnia-Hercegovina	4 852	326	1087 (919-1255)	5 055	179	684 (565-804)
Kosovo	2 838	91	1290 (646-1933)	2 226	37	971 (578-1364)
Middle East	***15 952***	***501***	***1026 (840-1211)***	***9 788***	***99***	***542 (389-694)***
Turkey	3 684	142	715 (552-877)	2 425	40	736 (405-1067)
Iraq	5 384	139	1229 (849-1609)	2 844	22	493 (215-771)
Iran	5 008	165	991 (714-1268)	3 427	30	510 (234-786)
North Africa	***4 122***	***73***	***630 (354-907)***	***1 847***	***9***	***136 (31-241)***
Morocco	2 277	43	590 (252-927)	1 223	5	116 (00-236)
Sub-Saharan Africa	***10 641***	***161***	***636 (444-828)***	***7 301***	***34***	***247 (150-343)***
Somalia	3 623	60	851 (324-1378)	2 575	9	170 (57-284)
South Asia	***13 491***	***916***	***1327 (1169-1485)***	***10 829***	***210***	***619 (511-727)***
Sri Lanka	3 760	135	1329 (786-1873)	3 045	25	453 (226-680)
India	2 560	131	895 (679-1111)	2 075	51	649 (461-838)
Pakistan	6 283	633	1533 (1306-1761)	5 171	131	646 (477-815)
Southeast Asia	***6 467***	***155***	***564 (436-692)***	***14 596***	***75***	***312 (223-402)***
Philippines	1 258	32	288 (183-393)	4 725	11	53 (13-93)
Vietnam	4 433	100	528 (383-673)	4 337	40	291 (191-391)
East Asia	***2 975***	***63***	***672 (493-852)***	***3 724***	***29***	***225 (140-311)***
China	1 949	51	679 (485-874)	2 216	18	196 (105-286)
Central-Asia	***1 382***	***39***	***1285 (344-2226)***	***1 235***	***12***	***668 (42-1294)***
North America	***6 976***	***377***	***768 (691-846)***	***7 746***	***336***	***355 (315-394)***
USA	6 089	351	791 (709-874)	6 724	288	331 (292-371)
Central-America	***731***	***19***	***927 (346-1508)***	***1 058***	***6***	***101 (15-187)***
South-America	***3 942***	***113***	***916 (594-1238)***	***4 495***	***45***	***292 (192-393)***
Chile	*2 500*	*76*	*476 (278-673)*	*2 071*	*18*	*216 (87-346)*
Oseania/Pacific	***769***	***15***	***1236 (511-1961)***	***636***	***6***	***164 (28-300)***

AMI: Acute Myocardial Infarction; SER: Standardized event rate; CI: Confidence Interval

Table A3 Age standardized stroke event rates per 100 000 person-years for subjects aged 35-89 years, CVDNOR 1994-2009

	Men 35-89 years, n=1 634 520			Women 35-89 years, n=1 682 539		
Country or region of birth	N	Strokes	SER (95 % CI)	N	Strokes	SER (95 % CI)
Norway	1 472 970	114072	744 (739-748)	1 542 459	109861	509 (506-512)
Western Europe	***59 931***	***1858***	***763 (723-804)***	***51 919***	***1990***	***495 (473-517)***
Denmark	11 830	696	846 (774-919)	11 004	535	492 (449-536)
Finland	3 384	125	847 (650-1043)	4 148	122	545 (439-651)
Sweden	13 191	356	727 (641-813)	13 139	510	501 (458-545)
The Netherlands	3 062	73	743 (524-962)	2 416	44	334 (230-438)
Great Britain	10 268	196	641 (520-762)	6 591	299	488 (433-543)
Germany	8 664	220	883 (748-1018)	7 390	266	505 (444-567)
Eastern Europe	***23 801***	***350***	***872 (773-971)***	***15 324***	***224***	***492 (423-562)***
Poland	16 083	159	924 (767-1081)	5 804	82	442 (330-553)
Russia	1 499	13	404 (172-636)	4 396	44	526 (350-702)
Hungary	938	82	952 (681-1223)	675	41	600 (404-796)
Former Yugoslavia	***10 370***	***290***	***725 (607-844)***	***9 582***	***249***	***653 (558-749)***
Bosnia-Hercegovina	4 852	182	711 (572-850)	5 055	179	666 (550-781)
Kosovo	2 838	29	867 (123-1610)	2 226	26	661 (372-951)
Middle East	***15 952***	***191***	***679 (493-865)***	***9 788***	***93***	***449 (320-578)***
Turkey	3 684	59	440 (263-617)	2 425	23	555 (233-876)
Iraq	5 384	65	676 (387-965)	2 844	37	566 (363-769)
Iran	5 008	52	682 (377-987)	3 427	27	351 (156-545)
North Africa	***4 122***	***40***	***437 (167-708)***	***1 847***	***17***	***465 (209-721)***
Morocco	2 277	15	114 (51-177)	1 223	10	347 (97-597)
Sub-Saharan Africa	***10 641***	***158***	***760 (542-977)***	***7 301***	***55***	***404 (273-534)***
Somalia	3 623	64	885 (566-1204)	2 575	13	201 (61-342)
South Asia	***13 491***	***295***	***655 (515-796)***	***10 829***	***200***	***628 (512-744)***
Sri Lanka	3 760	41	403 (200-606)	3 045	29	413 (216-610)
India	2 560	61	677 (405-948)	2 075	39	547 (364-731)
Pakistan	6 283	186	682 (494-870)	5 171	127	736 (542-929)
Southeast-Asia	***6 467***	***159***	***696 (550-841)***	***14 596***	***208***	***593 (480-706)***
Philippines	1 258	21	522 (62-983)	4 725	50	244 (139-348)
Vietnam	4 433	120	709 (547-870)	4 337	115	647 (510-784)
East Asia	***2 975***	***74***	***703 (530-876)***	***3 724***	***72***	***526 (398-654)***
China	1 949	59	700 (513-888)	2 216	47	488 (348-628)
Central-Asia	***1 382***	***8***	***308 (2-614)***	***1 235***	***15***	***1132 (234-2030)***
North-America	***6 976***	***317***	***643 (573-713)***	***7 746***	***427***	***478 (430-525)***
USA	6 089	285	638 (564-711)	6 724	381	479 (428-530)
Central-America	***731***	***18***	***886 (353-1419)***	***1 058***	***12***	***369 (81-657)***
South-America	***3 942***	***60***	***691 (376-1005)***	***4 495***	***69***	***472 (339-605)***
Chile	*2 500*	40	443 (94-791)	*2 071*	26	361 (184-539)
Oseania/Pacific	***769***	***7***	***633 (92-1174)***	***636***	***8***	***227 (68-387)***

SER: Standardized event rate; CI: Confidence Interval
